# Differences between Dyslexic and Non-Dyslexic Children in the Performance of Phonological Visual-Auditory Recognition Tasks: An Eye-Tracking Study

**DOI:** 10.1371/journal.pone.0159190

**Published:** 2016-07-20

**Authors:** Aimé Tiadi, Magali Seassau, Christophe-Loïc Gerard, Maria Pia Bucci

**Affiliations:** 1 UMR 1141 Institut National de la Santé Et de Recherche Médicale- Paris Diderot, Robert Debré Hospital, Paris, France; 2 e(ye) Brain, Ivry sur Seine, France; 3 Child and Adolescent Psychiatry Department, Robert Debré Hospital, Paris, France; Hangzhou Normal University, CHINA

## Abstract

The object of this study was to explore further phonological visual-auditory recognition tasks in a group of fifty-six healthy children (mean age: 9.9 ± 0.3) and to compare these data to those recorded in twenty-six age-matched dyslexic children (mean age: 9.8 ± 0.2). Eye movements from both eyes were recorded using an infrared video-oculography system (MobileEBT^**®**^ e(y)e BRAIN). The recognition task was performed under four conditions in which the target object was displayed either with phonologically unrelated objects (baseline condition), or with cohort or rhyme objects (cohort and rhyme conditions, respectively), or both together (rhyme + cohort condition). The percentage of the total time spent on the targets and the latency of the first saccade on the target were measured. Results in healthy children showed that the percentage of the total time spent in the baseline condition was significantly longer than in the other conditions, and that the latency of the first saccade in the cohort condition was significantly longer than in the other conditions; interestingly, the latency decreased significantly with the increasing age of the children. The developmental trend of phonological awareness was also observed in healthy children only. In contrast, we observed that for dyslexic children the total time spent on the target was similar in all four conditions tested, and also that they had similar latency values in both cohort and rhyme conditions. These findings suggest a different sensitivity to the phonological competitors between dyslexic and non-dyslexic children. Also, the eye-tracking technique provides online information about phonological awareness capabilities in children.

## Introduction

Developmental dyslexia is a specific impairment in learning how to read, despite of normal intelligence and education. It is well established that phonological abilities constitute the prerequisite to efficient reading [1, 2, 3]. Deficits in phonological awareness, the ability to identify and manipulate independently the syllables, phonemes or rhymes of a word are strongly linked to dyslexia [4, 5, 6]. Thus, one influential theory of dyslexia’s etiology, i.e., the phonological deficit hypothesis, suggests the impairments in the representation and processing of speech sounds as the cause of reading impairment in dyslexia [7, 8]. The speech sound impairments could be also in relationship with the low-level auditory processing observed in these children [9].

The methodologies traditionally used to measure phonological awareness in dyslexic children usually involve the manipulation of rhymes, syllables and phonemes. For example, Rack and collaborators [10] reported that English dyslexic children had difficulty in suppressing the initial or final phonemes and syllables. Wimmer [11] asked German dyslexic and non-dyslexic children to read words and non-words and replace the initial phoneme of monosyllabic and dissyllabic words in order to generate non-words. This author found that dyslexic children were impaired in non-word reading and exhibited more difficulties at replacing the initial phoneme than non-dyslexic children. In a cross-linguistic review (of English, French, German and Spanish languages), Sprenger-Charolles and collaborators [12] showed that the main difficulty of dyslexic children is to process automatically the rhymes, syllables and phonemes, and they argued that this difficulty varied with the transparency of the orthographic system.

Phonological awareness could also be objectively measured by using an eye-tracker to record the eye movements while children performing phonological tests. The eye-tracker technique also allows a real-time monitoring of eye movements during the visual-auditory recognition task. For instance, Jones and collaborators [13] recorded eye movements in dyslexic and non-dyslexic young children during a rapid naming task (RAN). They manipulated the rhyme, the onset and the visual letter sets in different conditions; in the confusable condition, phonological items were presented adjacently whereas in the non-confusable condition, in which the items were presented non-adjacently. In the non-confusable condition, the authors found longer fixation duration as well as longer visual processing time of the letters for dyslexic subjects than for non-dyslexic children; in contrast, in the confusable condition both groups of subjects had similar fixation durations. The authors suggested that both phonological and visual processes influence phonological naming-speed, but dyslexic subjects are more likely to suffer deficits in these processes.

The visual-auditory word recognition allows the evaluation of phonemic and rhyming awareness, together with eye movement recordings, have also been applied to the study of normal adult readers [14, 15, 16] as well as normal and dyslexic children [17, 18, 19]. For instance, Allopena and collaborators [14] recorded eye movements to examine the cohort and rhyme competitor effects on visual-auditory recognition in adults. They compared the fixation probability on targets in the presence of phonologically competiing objects and unrelated objects. These authors found that the probability to fixate both distracting objects that share a cohort or rhyme with the objects was higher, suggesting that they compete for object recognition. Meyer and collaborators [15] recorded the latency and direction of the primary saccade directions in adult subjects during a visual-auditory recognition task to measure the rhyme competitor effects. They found that the presence of rhyme competitors delayed the visual-auditory recognition responses and that the first saccade was more likely directed toward the rhyme competitor than toward the unrelated objects, suggesting that phonological competitors attract the subject’s attention in visual-auditory recognition. Görges and collaborators [16] replicated Meyer’s findings and also reported that the familiarity of the object and its name plays a role in visual-auditory recognition.

The developmental trend of the cohort competitor effect was first explored by Sekerina and collaborators [17]. They compared the number of fixation and the latency of the first saccade to cohort-related and cohort-unrelated pictures in Russian children (5 to 6 years old) and adults. They showed that, unlike adults, children had the same number of fixations on cohort-related and -unrelated pictures, suggesting the cohort competitor effect was absent in children. However, they found a difference in saccade latency to these two types of pictures in children, but not in adults, establishing that in children the phonological competitor effect appear to exist only in the early stages of reading development.

Desroches and collaborators [18] tested the phonological competitor effects using an eye-tracker in a visual-auditory word recognition task in a small group of dyslexic and control children (eight dyslexics and nine control children of about 9 years old). In this study the target object was presented at the same time as three distractors. In the baseline condition, all of the three distractors were phonologically unrelated items; in the rhyme condition, one item was a rhyme distractor; in the cohort condition, one item was a cohort distractor (word with initial monosyllable or syllable); and in the rhyme+cohort condition, there were both rhyme and cohort distractors. The results showed that the speed and accuracy of word recognition as comparable across dyslexics and healthy controls in the baseline condition. While non-dyslexic children were sensitive to rhyme and cohort competitors, showing low probability of fixating the target object when distractors were present, dyslexic children showed a cohort competitor effect only.

In the present study, we examined oculomotor behaviors in a task similar to that used by Desroches and collaborators, but had a larger group of healthy children (fifty-six) to map out the developmental trend of competitor effects in visual word recognition. Furthermore, we compared oculomotor behaviors of healthy children to that of dyslexic children (twenty-six) to clarify whether the oculomotor behaviors are impacted when rhyme processing is lacking, as in dyslexia [17].

## Methods

We conducted two experiments in this study. The aim of the first experiment was to explore the developmental trend in healthy children by analyzing the oculomotor parameters and the phonological competitor effects that could indicate the level of phonological awareness of different age subgroups of healthy children. The aim of the second experiment was to investigate the visual-auditory recognition capabilities between healthy children and dyslexic children through the analysis of the same phonological competitor effects.

### Subjects

The investigation adhered to the principles of the Declaration of Helsinki and was approved by our Institutional Human Experimentation Committee (CPP Ile de France I). Written consent was obtained from the children's parents after they were given an explanation about the experimental procedure.

In Experiment 1, fifty-six healthy children from 6 to 13 years old (mean age: 9.9 ± 0.3) participated in the study. For easy presentation of these children, they were divided into four groups: eleven children between 6 and 7 years old (mean age: 6.9 ± 0.3; 4 females and 7 males), eighteen children between 8 and 9 years old (mean age: 8.7 ± 0.7; 9 females and 9 males), sixteen children between 10 and 11 years old (mean age: 11.2 ± 1.09; 9 females and 7 males) and eleven 12- to 13-years old (mean age: 13.3 ± 0.5; 8 females and 3 males). ANOVA test of the mean age showed a significant difference between all these sub-groups (F _(3,51)_ = 542.26, p < 0.001). The inclusion criteria were as follows: no known neurological or psychiatric abnormalities, no history of reading difficulty, no visual impairment, or difficulty with near vision. A self-made questionnaire was completed by parents before the inclusion of their child. Both the similarity test of the WISC IV (assessing their verbal capability by abstracting criteria common to two objects and by excluding the differences, which requires adequate cognitive functioning), and the matrix test of the WISC IV (assessing their logic capability) were performed by a neuropsychologist. Normal range for both tests is 10 ± 3 (Wechsler intelligence scale for children—fourth edition, 2004). All the non-dyslexic children tested (see Table 1) had both normal verbal capabilities (11.9 ± 0.3) and normal logic (11.4 ± 0.3).

**Table 1:** Clinical characteristic of the groups of children examined in the experiment 1 and experiment 2.

Clinical characteristics	Experiment 1	Experiment 2	
	Non-dyslexic children	Non-dyslexic children	Dyslexic children
	(N = 56)	(N = 34)	(N = 26)
Chronological age (yrs)	9.9 ± 0.3	9.6 ± 0.2	9.8 ± 0.2
Reading age (yrs)	9.8 ± 0.1	9.4 ± 0.3	7.5 ± 0.2
Verbal IQ			100 ± 1.3
Verbal Sc	11.9 ± 0.3	11.5 ± 0.4	
Logic IQ			99 ± 1.5
Logic Sc	11.4 ± 0.3	11.8 ± 0.4	

In order to know whether there are some differences between healthy children and dyslexics in the performance of phonological visual-auditory recognition task, we achieved a second experiment (Experiment 2). Thus, thirty-four chronological age-matched non-dyslexic children (mean age: 9.6 ± 0.2) selected among the healthy children from Experiment 1 were compared with twenty-six dyslexic children from 8 to 11 years old (mean age: 9.8 ± 0.2). Clinical characteristics of all the children who participated in this study are shown in Table 1. Note that the number of dyslexic children was smaller with respect to that of non-dyslexic children because eight dyslexic children withdrew from the experiment given that they did not achieve the full experiment and, therefore, their data were incomplete.

In Experiment 2, both groups of children were separated into two subgroups: thirteen dyslexic children and eighteen non-dyslexic children from 8 to 9 years old, twelve dyslexic children and sixteen non-dyslexic children from 10 to 11 years old. Normal children were selected based on their reading capabilities assessed by the ELFE test (cogni-sciences, Grenoble). Dyslexic children were recruited from Robert Debré pediatric hospital, to which they had been referred for a complete evaluation of their dyslexia with an extensive examination including neurological/psychological and phonological capabilities. For each child, we measured the time they required to read a text passage, assessed their general text comprehension, and evaluated their ability to read words and pseudo-words using the L2MA battery [20]. This is the standard test developed by the Centre de Psychologie appliquée de Paris, often used in France and already employed in our previous studies for selecting dyslexic populations [21, 22, 23, 24, 25]. In France, a child is considered to be dyslexic when her/his reading capabilities are delayed at least beyond 1.5 standard deviations with respect to age-matched children. As shown in Table 1, ANOVA reported a significant difference for reading age between the two groups of children who participated in Experiment 2.

### Stimuli

The paradigm was similar to that used by Desroches and collaborators. We selected the words by using the “Lexique.org” base, which is a French database allowing one to find words having common prefixes and rhymes. The frequency of each word was extracted from http://www.manulex.org/fr/infra/results.html?page=95&fullscreen=1, a French site which provides a lexicon database and the written word frequencies from first to fifth grades. The words were chosen in order to give responses to the various experimental conditions: at least one common prefix with another word; at least one common rhyme with another word; and having some phonological unrelated distractors in common. In sum 29 items were selected as target words, and each item was presented as a target word in the four experimental conditions. The target word was also presented as a distractor in order to avoid any learning bias.

**Fig 1:**
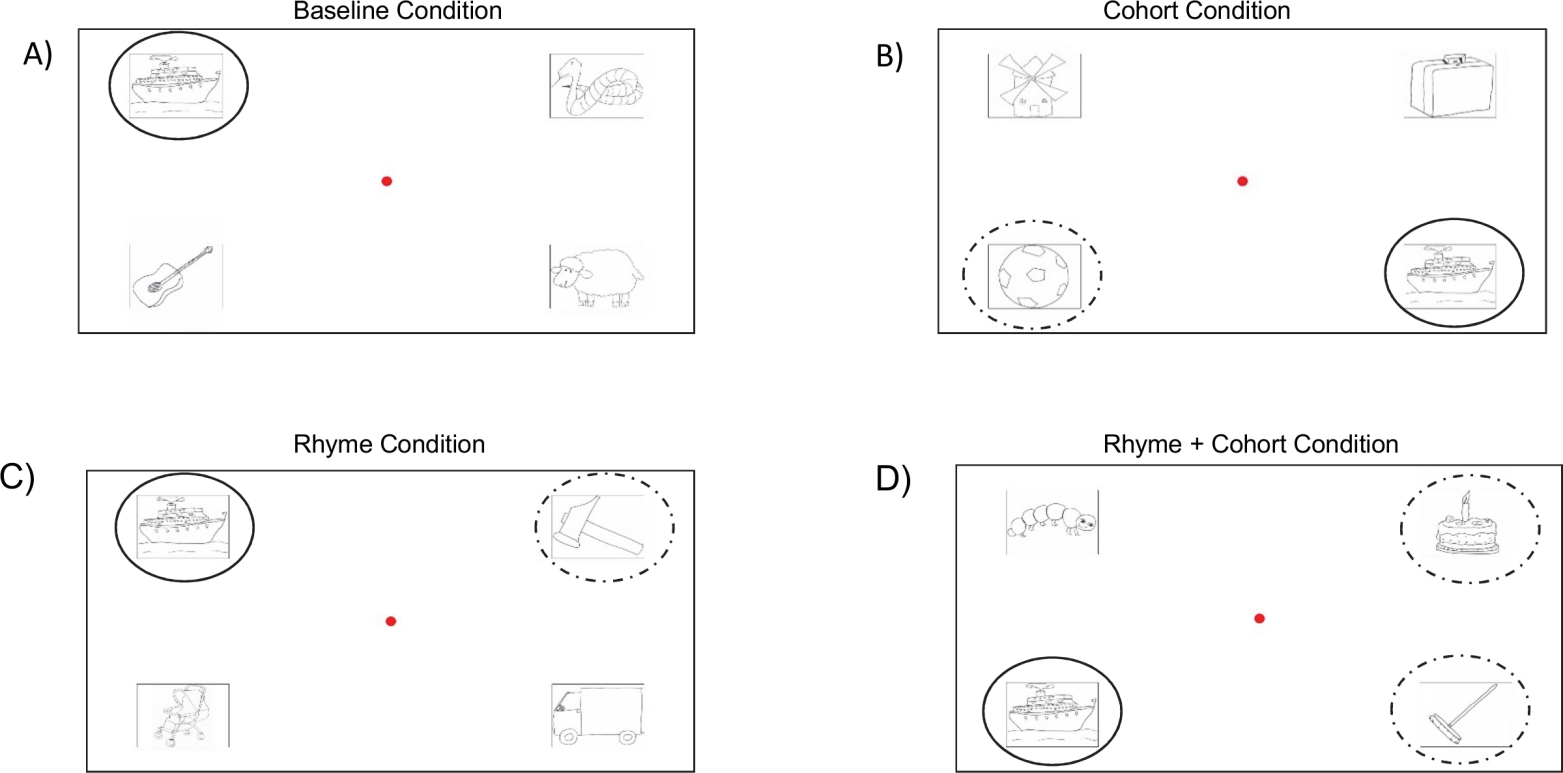
Presentation of the four experimental conditions for the target word « bateau ». A) Baseline condition: the target word was presented at the same time of other three phonological unrelated objects (serpent, guitare, vache); B) cohort condition: « ballon » is the cohort competitor object together with other two unrelated objects (maison, cartable); C) rhyme condition: « marteau » is the rhyme competitor object together with two unrelated objects (poussette, voiture); D) rhyme + cohort condition: « gâteau » and « balai » are rhyme and cohort competitor objects together with one unrelated object (chenille).

Then, we chose the corresponding stimuli. The stimuli were objects drawn with a pencil on a black and white background. They were presented on a 22-inch PC screen with a resolution of 1920×1080 and a refresh rate of 60 Hz. A total of 116 scenes divided into 3 blocks were presented to the children in order to allow them to take some breaks. Fig 1 represents the four experimental conditions for the target word « bateau ». In the baseline condition (Fig 1A), there is the target object and three phonological unrelated objects. The PC’s screen was 50 cm x 32 cm and stimulus size was 6° x 5°. In the cohort condition (Fig 1B), « ballon » is the cohort competitor object together with two unrelated objects; in the rhyme condition (Fig 1C), « marteau » is the rhyme competitor object together with two unrelated objects; in the « rhyme + cohort » condition (Fig 1D), « gâteau » and « balai » are rhyme and cohort competitor objects together with one unrelated object. The items were presented randomly. An example of word frequency is shown in Table 2 for the target object ‘bateau’.

**Table 2:** Example of Words’ Frequency and their appearance in each condition tested for the target word “Bateau”.

Words	Frequency (in millions of occurrences)	Baseline	Cohort	Rhyme	Rhyme + Cohort
**Balai**	108.18	**-**	**-**	**-**	**+**
**Baleine**	72.93	**-**	**-**	**-**	
**Ballon**	216.64	**-**	**+**	**-**	**-**
**Bateau**	190.37	**+**	**+**	**+**	**+**
**Cadeau**	189.54	-	+	-	-
**Camion**	161.05	-	-	+	-
**Chenille**	10.71	-	-	-	+
**Guitare**	189.79	+	-	-	-
**Marteau**	53.99	-	-	+	-
**Mouton**	172.46	+	-	-	-
**Serpent**	78.97	+	-	-	-

The sign (-), means that the object did not appear in the condition.

The sign (+), means that the object appeared in the condition.

Prior to the experiment, the children had to name each drawing presented alone. The correct response was provided to the child when he/she made an error and the test started when the child was able to recognize all the drawings. All the drawings were presented in a framework of 300 x 230 pixels. They appeared randomly in the four angles of the screen respectively on the horizontal and vertical plans to -16.8° / 8.9°; 16.8° / 8.9°; -16.8° / -8.9°; 16.8° / -8.9°. Each scene comprised four drawings. We asked the children to fixate a cross in the center of the screen during 2 seconds. Then, the four drawings appeared at the same time and the children had to look at them freely during 3 seconds. A computer sound indicated them to fixate the central point again. Next, the target word was presented auditorily and the children had to fixate the corresponding drawing during a period of 3 seconds.

### Eye movement recordings

Eye movements were recorded with the Mobile Eyebrain Tracker (Mobile EBT^**®**^, e(ye)BRAIN, www.eye-brain.com), an eye-tracking device CE for medical purposes. The Mobile EBT^®^ benefits from a high frequency camera that allows it to record both the horizontal and vertical eye positions independently and simultaneously for each eye. Recording frequency was set up to 300 Hz. The precision of this system is 0.25°. The recording system does not obstruct the visual field, and the calibrated zone covers a horizontal visual angle of ± 22° [26].

A calibration for eye movement recordings was conducted before the stimulus presentation. During the calibration procedure, the children were asked to fixate a grid of 13 points (diameter 0.5 deg) mapping the screen. Point positions in degree in horizontal/vertical plans were: -20.9°/12.2°, 0°/12.2°, 20.9°/12.2°, -10.8°/6.2°, 10.8°/6.2°, -20.9°/0°, 0°/0°, 20.9°/0°, -10.8°/-6.2°, 10.8/-6.2°, -20.9°/-12.2°, 0°/-12.2°, 20.9°/-12.2°.

Each calibration point required a fixation of 250 ms to be validated. A polynomial function with five parameters was used to fit the calibration data and to determine the visual angles. After the calibration procedure, the children started the experiment.

### Data processing

The software MeyeAnalysis (provided with the eye tracker, e(ye)BRAIN) was used to extract saccadic eye movements from the data. It determines automatically the start and the end of each saccade by using a built-in saccade detection algorithm. The algorithm used to detect saccades is adapted from [27]. The algorithm searches for velocity peaks by identifying samples where the velocity is larger than a velocity threshold (Ɵ > Ɵ_PT_). An iterative data-driven approach is proposed to find a suitable threshold. The iterative algorithm is given an initial peak velocity detection threshold PT_1_, which could be in the range 100°-300°/sec, but the choice is not critical as long as there are saccades with peak velocities reaching this threshold [28].

Then, each saccade was treated according to the pre-defined Region Of Interest (ROI). The ROI corresponded to the position of the drawings on the screen, *i*.*e*. four regions of 300 x 230 pixels positioned to—16.8° / 8.9°; 16.8° / 8.9°; -16.8° / -8.9°; 16.8° / -8.9°.

Two parameters were measured for each ROI. First, the percentage of the total time spent on the target during the 3-sec free viewing. Secondly, we measured the latency of the first saccade oriented on each target. This was done in order to complete Desroches’s study, in which the fixation rate on the target was the main variable tested for exploring speed and accuracy of the participants’ reactions. Also, we wanted to measure how the effect of visual distractors on the saccade latency, as reported previously [38], could affect the visual-recognition of both control and dyslexic children.

### Statistical analysis

ANOVA was performed with the Statistica software. In Experiment 1, we used two-way ANOVA to analyze the main condition effect and age effect for the different groups of children. In Experiment 2, we used the two groups of children (dyslexic and non-dyslexic) as inter-subject factor and the four conditions and age ranges as within-subject factors. *Post-hoc* comparisons were made with the Fischer’s test (LSD). The effect of a factor was considered as significant when the p-value was below 0.05.

## Results

### Experiment 1

#### Percentage of the total time spent on the target

**Fig 2:**
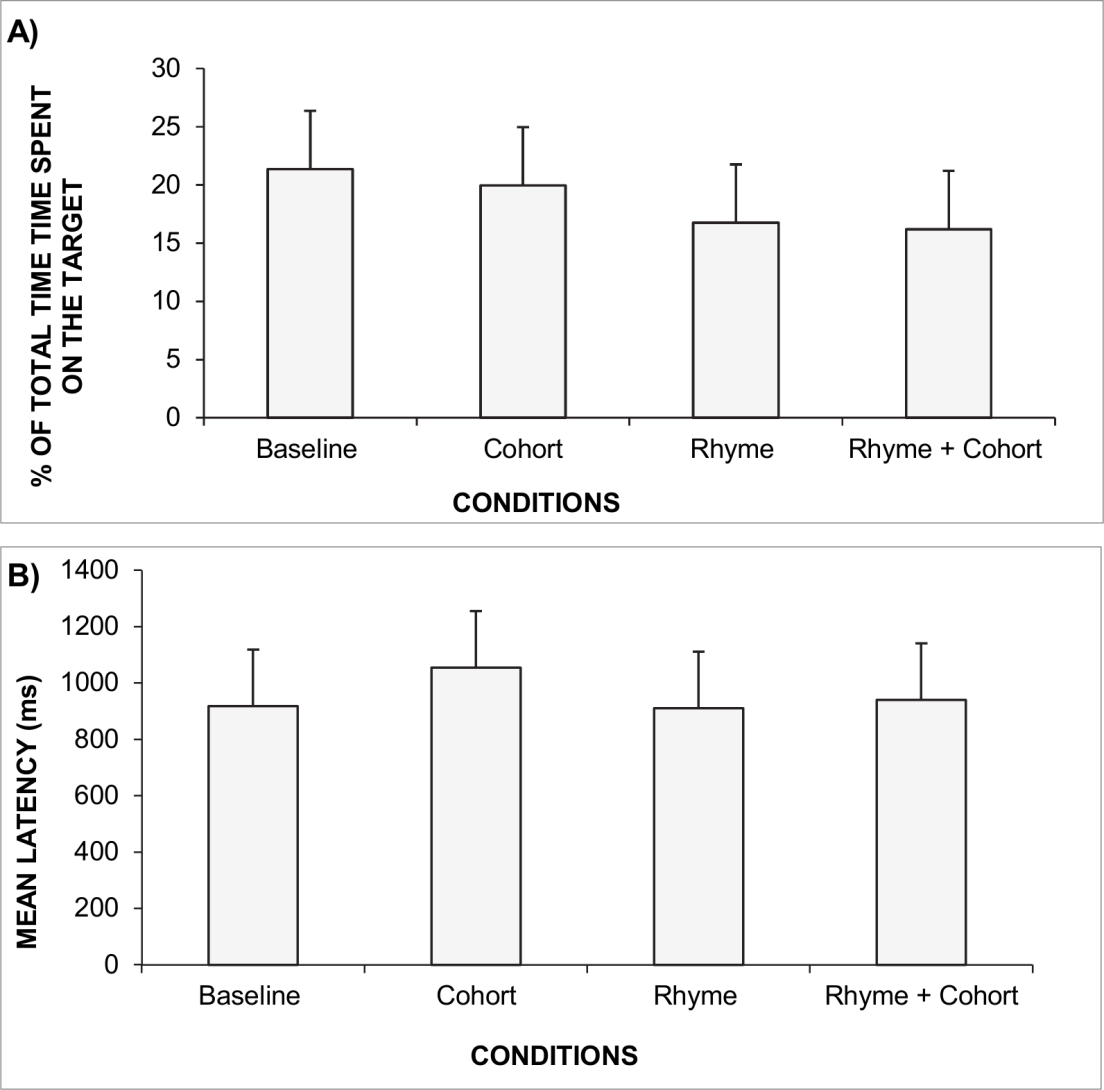
Mean of the percentage of the total time spent in the target in each condition tested (baseline, cohort, rhyme and rhyme + cohort) for non-dyslexic children (A); Mean latency (in ms) of the first saccade in each condition tested (baseline, cohort, rhyme and rhyme + cohort) for non-dyslexic children (B). Vertical bars indicate standard errors.

Fig 2A shows the percentage of the total time spent on the target by all the healthy children tested. ANOVA shows a main effect of condition (F_(3,156)_ = 3.443, p<0.01). The *Post-hoc* test shows that in the baseline condition the percentage of the total time spent on the target was significantly larger than that spent in the rhyme condition (p<0.002) and in the rhyme + cohort condition (p<0.002); and also that the percentage of the total time spent on the target was significantly larger in the cohort condition with respect to the rhyme + cohort condition (p<0.04). ANOVA failed to show a significant effect of age (F_(3, 52)_ = 1. 85, p = 0. 15) and did not reveal any interaction between condition and age (F_(9,156)_ = 1.15, p = 0. 33). The mean percentages of total time spent on the target are shown in Table 3.

**Table 3:** Mean total time spent on the target and mean latency in non-dyslexic children in experiment 1.

Age ranges	Conditions	Non-dyslexic children	
		Mean % of total time	Mean latency (ms)
	
6–7 years (6.9 ± 0.3)	Baseline	14.98	957.74
	Cohort	11.42	994.26
	Rhyme	12.17	875.89
	Rhyme + Cohort	10.65	918.81
8–9 years: (8.7 ± 0.7)	Baseline	22.60	970.68
	Cohort	21.37	1017.90
	Rhyme	12.96	997.10
	Rhyme + Cohort	17.04	938.83
10–11 years (11.2 ± 1.09)	Baseline	24.13	830.42
	Cohort	25.46	1077.99
	Rhyme	20.51	909.74
	Rhyme + Cohort	17.43	889.33
12–13 years (13.3 ± 0.5)	Baseline	21.69	780.43
	Cohort	18.20	900.89
	Rhyme	21.40	834.44
	Rhyme + Cohort	19.73	756.30

#### Latency of the first saccade

Fig 2B shows the mean latencies of the first saccade in each condition for all the healthy children tested. ANOVA shows a main effect of condition (F_(3,156)_ = 4.092, p<0.007). *Post-hoc* tests reveal that the latency of the first saccade in the cohort condition was significantly longer with respect to the baseline condition (p<0.002), the rhyme condition (p<0.004) and also the rhyme + cohort condition (p<0.01).

ANOVA also showed a significant age effect (F_(3, 52)_ = 9.464, p<0.00004): the latency decreased significantly as age increased. *Post-hoc* tests showed that 6–7 years old children had significantly longer latencies than the 10–11 years *(*p<0.0003) and 12–13 years old children groups (p<0.00005). In the same way, the 8–9 years old group had significantly longer latencies than 10–11 and 12–13 years old children (p<0.004 and p < 0.0005, respectively). All values are shown in Table 3. ANOVA failed to show an interaction between condition and age (F_(9, 156)_ = 1.13, p = 0.34).

### Experiment 2

#### Percentage of the total time spent on the target

**Fig 3:**
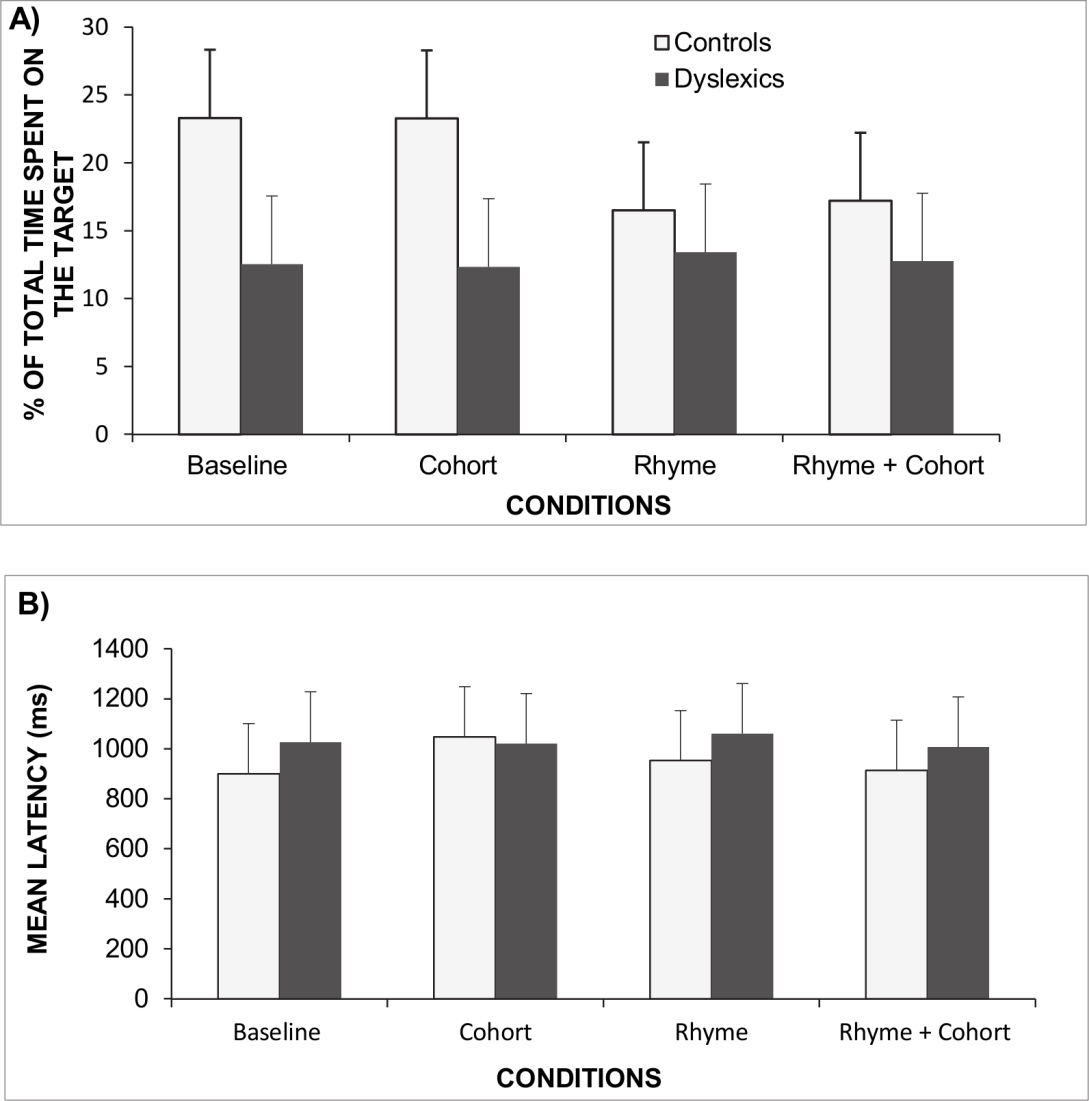
Mean of the percentage of the total time spent in the target in each condition tested (baseline, cohort, rhyme and rhyme + cohort) for dyslexic and non-dyslexic children (A); Mean latency (in ms) of the first saccade in each condition tested (baseline, cohort, rhyme and rhyme + cohort) for the dyslexic and non-dyslexic children (B). Vertical bars indicate standard errors.

Fig 3A shows the percentage of the total time spent on the target for both non-dyslexic and dyslexic children in all conditions tested. ANOVA showed a significant group effect (F_(1, 55)_ = 8.707, p<0.004): the total time for the control children group was significantly longer than for the dyslexic children group.

ANOVA also showed a significant effect of condition (F_(3,165)_ = 2.896, p<0.03); a *post-hoc* test showed that the percentage of total time spent in baseline condition was significantly longer than the time spent in the rhyme and the rhyme + cohort conditions (both p<0.01); the percentage of total time spent in the cohort condition was also significantly longer than that spent in the rhyme and in the rhyme + cohort conditions (both p<0.01).

ANOVA also revealed a significant interaction between group and condition (F_(3,165)_ = 4.32, p<0.005); a *post-hoc* test showed that for non-dyslexic children the percentage of the total time spent in the baseline and cohort conditions was significantly longer than those reported for dyslexic children in all conditions tested (all p<0.0001). Unlike dyslexics, age-matched non-dyslexic children also showed significantly shorter total time in rhyme condition compared with baseline condition (p<0.0002) and cohort condition (p<0.0002). ANOVA did not show any effect of age and did not reveal any interaction between group, condition and age (F_(3,165)_ = 1.44, p = 0.23). The mean percentages of total time spent on the target are shown in Table 4.

**Table 4:** Mean total time spent on the target and mean latency in both non-dyslexic and dyslexic groups in experiment 2.

Age ranges	Conditions	Non-dyslexic group		Dyslexic group	
		Mean % of total time	Mean latency (ms)	Mean % of total time	Mean latency (ms)
	
8–9 years (8.7 ± 0.7)	Baseline	22.6	970.68	11.34	1042.32
	Cohort	21.37	1017.9	12.09	1041.35
	Rhyme	12.96	997.1	14.05	1078.16
	Rhyme + Cohort	17.04	938.83	12.05	994.23
10–11 years (11.2 ± 1.09)	Baseline	24.13	830.42	13,86	1013.09
	Cohort	25.46	1077.99	12,64	1000.91
	Rhyme	20.51	909.74	12.76	1043.41
	Rhyme + Cohort	17.43	889.33	13.54	1021.12

#### Latency of the first saccade

Fig 3B shows the mean latencies of the first saccade for both groups of children (non-dyslexics and dyslexics) in all conditions tested. ANOVA showed a significant group effect: dyslexic children were slower than non-dyslexic children (F_(1,55)_ = 5.568, p<0.02). ANOVA did not find a significant effect of conditions, but a significant effect of age (F _(1, 55)_ = 4. 760, p<0.03). The *post-hoc* tests revealed that 10–11 years old non-dyslexic children had a significantly shorter latency than the other two dyslexic children groups (8–9 years old and 10–11 years old group, p<0.002 and p<0.004, respectively). Finally, ANOVA did not reveal a significant interaction either between group and condition (F _(3,165)_ = 1.39, p = 0.24) or between group, condition and age (F _(3,165)_ = 5.26, p = 0.66). The mean latency values are shown in Table 4.

## Discussion

The main findings of the first experiment on healthy children are as follows: (1) Developmental improvement of latency and phonological awareness; (2) The competitor effect of cohort and rhyme objects. The main difference reported in the second experiment comparing dyslexic with non-dyslexic children is: (3) Dyslexic children are not influenced by competitors; (4) The attentional difficulties in dyslexic children could explain their smaller total time spent on the object-target. These findings are discussed individually below.

### Developmental improvement of latency and phonological awareness

The present study shows that the latency decreased with the increasing age of the children independently from the conditions tested. This is not surprising because previous studies, conducted on the developmental aspect of eye movements by our team [29, 30] as well as by other researchers [31, 32, 33], showed that latency of eye movements improved with age of children and reached adult level at about 12 years old. Indeed, Hutton [34] as well as McDowell [35] described the presence of distinct cortical circuits responsible for eye movement’s preparation and Luna and collaborators [36] reported that such brain structures are not completely developed in children. As shown by Gogtay [37] and Toga [38], the reduction in grey matter in the frontal and temporal areas occurs throughout childhood until adolescence.

Moreover in this study we reported a developmental trend of the phonological awareness; indeed visual-auditory recognition was faster in children between ten and thirteen years old, maybe because of a better development of phonological representation of the target object reported in other studies which, however, did not record eye movements [39, 40, 41]. In English children, Goswani and Bryant [39] showed that phonological awareness developed progressively from global syllable awareness to phoneme awareness; younger children first developed global rhyme and syllable awareness, and phoneme awareness afterwards. Through a longitudinal study in English children, Berninger and collaborators [40] explored the developmental trend of different components of linguistic awareness in a large population of children from first to sixth grade in order to establish the relationship between linguistic awareness and reading skills. These authors found that phonological awareness improved with age of children and interacted with orthographic and morphologic awareness when reading. Bentin and collaborators (1991) [41] measured the effects of aging and schooling on the development of phonological awareness in Hebrew children from five to seven years old and showed that school had a more significant effect than aging on phonological awareness development, most likely because at school children could develop formal reading abilities given by teachers. In Spanish language, Carillo [42] also reported that children between six and seven years old had better phonological awareness than those from five to six years old.

Sprenger-Charolles and collaborators [43] conducted a longitudinal study on the development of phonological and orthographic skills during reading, spelling and orthographic tasks in French children from six to ten years old. The results showed significant effects of age in all tasks tested and also reported that phonological skills are still developing in children until about ten years old. These authors, in line with previous studies in English populations, advanced the hypothesis that phonological and orthographic capabilities were related to the acquisition of reading skills.

Our findings confirm and enlarge the development of phonological awareness through a visual-word recognition task using an eye-tracker in French healthy children from six to thirteen years old. Indeed, this study can give insights on developmental trend of phonological awareness revealed online and rapidly by the use of oculomotor recordings. Recall that eye-tracking advantages were previously reported for reading development as well as online cognitive processes underlying eye movements [44, 45]. For example, in a review, Miller and O’Donnell [45] reported that eye movements’ data constituted an efficient indicator of online processing during reading.

### Competitor effect of cohort and rhyme

The present study showed that healthy children spent a significantly longer time on the object-target in the baseline condition than in the other conditions, suggesting that healthy children are sensible to phonological competitors. This could be explained by the fact the time spent on object-target is shortened because children were attracted by phonological competitors and, therefore, shared the object-target recognition time with those of the cohort and rhyme objects. This finding is in line with the phonological competitors’ effect already reported by Desroches and collaborators’ study [18] on English healthy children, which measured the number of fixations in the presence of both cohort and rhyme competitors. These authors found fewer fixations in the rhyme and cohort conditions with respect to baseline condition, suggesting that healthy children encode the phonological relationships among the words during the visual-auditory recognition processing.

The present study reported also for the first time that children are slower in the cohort conditions with respect to the other conditions. The significantly longer latencies reported in healthy children in the cohort condition suggest a stronger competitor effect for cohort than for rhyme. This finding has already been reported in English adult subjects by Allopena and collaborators [14], who recorded eye fixations only. Taken together, all these findings suggest that normal subjects (children as well as adults) are more sensitive to cohort information than to rhyme information in visual-word recognition.

### Dyslexic children are not influenced by competitors

We observed that, contrary to non-dyslexic children, dyslexic children spent a similar total time in all conditions, suggesting that they are not influenced by phonological competitors. This could be due to a weaker phonological awareness in dyslexic children, leading to a difficulty at distinguishing the differences between the phonologically-related objects. Several studies have already clearly established this phonological deficit in dyslexic children [46, 47, 48, 49]. We also found that dyslexic children did not exhibit any significant difference in cohort and rhyme competitor conditions, while non-dyslexic children spent less time in the rhyme condition; we suggest that this different oculomotor behavior could be due to the fact that dyslexics are less sensitive to rhyme competitors than non-dyslexic subjects. This finding is in agreement with the study of Desroches and others suggesting that dyslexic children are less sensitive to the rhyme than to the phoneme in contrast to non-dyslexic children who are sensitive to both units of the word [50].

The similar oculomotor behavior of dyslexic children in all conditions could also be linked to both visual and auditory cortical structure deficits already reported in dyslexic children [51]. Clark and collaborators showed by MRI that the visual and auditory cortical areas were thinner in children with dyslexia, suggesting that these reduced sensory cortical structures could explain the difficulties of dyslexic children at processing both visual and auditory information. Based on such finding we postulated that the visual-auditory information processing for phonological competitors could not work correctly in dyslexics, leading to poor visual-auditory recognition abilities.

Importantly, for the first time, we found that for dyslexic children the latency of the first saccade was similar in all conditions tested; meaning that for them the recognition speed was not different in the presence of cohort or rhyme competitors.

Moreover, we also found that the latency of dyslexics was longer than that of non-dyslexic children. This could be in relationship with slower latency of saccades already reported in dyslexic children [25]. The fact that dyslexics were slower than non-dyslexics even in the baseline condition reveals that in both groups of children, recognition speed is different; this result contrasts with those of Desroches showing that the fixation of dyslexics and non-dyslexic children was similar, at least in the baseline condition. We think that this difference between Desroches’ study and the present work could be due to the different ages of the children examined in the two studies. Indeed, Desroches’ study examined children of 8–9 years old; and for this age range, in our study as in Desroches’, the children did not show any difference in their latency values. The significant difference of the latencies in our study was reported for dyslexic and non-dyslexic children of 10–11 years old only. This is not surprising given that the maturation of cortical structures responsible for saccade preparation is improving during childhood as suggested by Luna and collaborators [36] leading to a large variability of latency values before the age of 10–11 years. This could explain why we reported latency difference after this age only.

### The fact that dyslexic children spent significantly shorter time on the targets may be due to their attentional difficulties

We reported that dyslexic children spent less time on the object-target than non-dyslexic children in all the conditions tested, maybe because of poor attentional capabilities of dyslexic children, as has been already reported [52]. Note that the visual-attention processing is controlled by the cortical areas, particularly in the parietal cortex, and they receive information via the cells of the magnocellular pathway [53, 54, 55]. Both magnocellular pathway and posterior parietal cortex are believed to be impaired in dyslexic children and this leads to a deficit of visual spatial-attention processing [56, 57].

## Limitations

In the present study, we did not analyze the effect of word and syllable frequency on phonological visual-auditory recognition performance. Studies exploring such an issue will be useful in order to know further phonological visual-auditory capabilities in healthy as well as dyslexic children.

## Conclusion

In this study, we address the question of phonological competitor effects comparing non-dyslexic and dyslexic children through a visual-auditory recognition task. Our findings give some evidence about the correlation between phonological visual-auditory recognition and age for healthy children as well as developmental trend of phonological awareness. Importantly, the cohort and rhyme competitors do not have the same effects in dyslexic and in healthy children, leading to different performances; indeed, the sensitivity to phonological competitors is similar in dyslexic children.

Based on all these findings, we suggest that eye-tracking method is a reliable tool to assess a reader’s phonological capabilities and to measure the developmental trend of phonological awareness in healthy children. It could also be useful to strengthen the diagnosis of dyslexic children concerning their phonological skills. We could also advance the hypothesis that dyslexic children could benefit from reeducation using both cohort and rhyme conditions in order to improve their visual-auditory recognition capabilities. Further studies are needed to test such a hypothesis.
